# Longitudinal trajectory of gross motor skills in school-aged children with Rett syndrome

**DOI:** 10.3389/fneur.2025.1702703

**Published:** 2026-01-29

**Authors:** Anne-Marie Bisgaard, Kingsley Wong, Anne-Katrine Højfeldt, Michelle Stahlhut, Jenny Downs

**Affiliations:** 1 Department of Paediatrics and Adolescent Medicine, Center for Rett Syndrome, Rigshospitalet, Copenhagen, Denmark; 2 The Kids Research Institute Australia, University of Western Australia, Perth, WA, Australia; 3 Center for Clinical Research and Prevention, Bispebjerg and Frederiksberg Hospital, Copenhagen, Denmark; 4 Curtin School of Allied Health, Faculty of Health Sciences, Curtin University, Perth, WA, Australia

**Keywords:** children, determinants, gross motor skills, longitudinal study, Rett syndrome

## Abstract

**Background:**

In children with Rett syndrome, this study aimed to (1) describe gross motor skill trajectories; and (2) analyse the influences of genetic variant and comorbidities.

**Methods:**

This was a prospective longitudinal study conducted at the Danish National Center for Rett Syndrome 2008 to 2022. The Rett Syndrome Gross Motor Scale (RSGMS) was administered, and clinical data collected at each visit. Mixed-effects linear regression models were used to analyze the effects of age, genetic variant and comorbidities on gross motor skills. Clinical records data were reviewed.

**Results:**

Data for 33 children with a mean age of 7.3 years (SD 1.2) at first visit were followed for a mean duration of 6.8 years (SD 2.1). The mean RSGMS score was 24.0 (SD 13.2, total of 45) at baseline. Adjusting for age and genetic variant, all severity levels of epilepsy, autonomic breathing dysfunction and scoliosis, except for surgically corrected scoliosis, were associated with a 4-point decrease in RSGMS score every 5 years. Acute escalation of seizures, change in muscle tone, orthopedic surgeries and bone fracture could be associated capacity to maintain gross motor skills.

**Conclusion:**

We identified decline in gross motor skills during childhood. This novel natural history data can assist with interpretation of changes in gross motor skills following the administration of new therapeutics.

## 1 Introduction

Rett syndrome (RTT) is a rare neurodevelopmental disorder occurring in 1 in 9,000 liveborn females (1). The incidence in males is rarer (2). As with many rare diseases, there is a genetic origin and symptoms are chronic and complex (3). RTT is caused by a pathogenic variant on the *MECP2* gene (4). Following the first six-18 month of life where there may have been subtle signs of a developmental disease, RTT is characterized by loss of previously acquired hand and/or communication skills, and the development of altered gait and hand stereotypies (5–7). Altered neurodevelopment persists and is usually accompanied by comorbid health conditions such as scoliosis, sleep disruptions including insomnia, poor growth, autonomic dysfunction and epilepsy (8).

Knowledge of the genetic cause of rare diseases has accelerated the possibility of developing disease-modification precision medicines that hope to substantially reduce or even cure the condition. Increasingly, rare diseases will be treated with therapies that target the genes, protein products, and molecular pathways associated with the genetic variant (9). For RTT, two gene therapy programs are being conducted by Taysha Gene Therapies (NCT05606614, NCT06152237) and Neurogene (NCT05898620). The success of these and other upcoming trials partly depends on a detailed understanding of the disease and its natural history and having valid and reliable outcome measures (10, 11).

Evidence is accumulating through empirical observations in the clinic and observational research studies of gross motor decline in RTT (12, 13). Foley et al. reported 70 individuals over a three- to four-year period for whom Rett Syndrome Gross Motor Scale (RSGMS) scores were slightly decreased in 60% (12). In a longitudinal study with a duration of six to 12 years, we examined gross motor skill trajectories in 24 adults using the RSGMS (13, 14). Scores declined 3.4 points (total score 45 points) for every five-year increase in age and decline was more apparent with epilepsy and severe scoliosis that had been conservatively managed (13). The trajectory of gross motor skills in children with RTT over a similarly longer period has not been analyzed.

Many associated neurological conditions in RTT have their onset during childhood with symptoms including seizures, movement disorders, breathing disturbances and scoliosis (15–17). In adults with RTT, seizures and severe conservatively managed scoliosis, but not breathing disturbances, were associated with declines in gross motor skills (13). It is well known that muscle tone in RTT changes from hypotonia in early childhood to hypertonia, dystonia, and rigidity when older which could impact gross motor skills (18), yet this has not been examined in relation to change in gross motor skills. Because of disease complexity, there may be exacerbations of the respiratory health or seizures status, consistent with other complex conditions. To our knowledge, movement disorders and exacerbations in health status in RTT have not been examined in relation to gross motor skills in longitudinal studies.

This study examined the trajectory of gross motor skills measured by the RSGMS and coded from video recordings in Danish school-aged children with RTT (14). Consistent with our previous study of adults (13), we evaluated associations with changes in the following variables: epilepsy, breathing disturbance, scoliosis, *MECP2* variant and/or age. We reviewed medical records at Rigshospitalet in Denmark to explore if other changes in health status could explain changes in gross motor skills.

## 2 Methods

### 2.1 Study design and population

This was a retrospective longitudinal study evaluating data collected in the Danish RTT database (13) and videos from clinical visits at the Danish National Center for Rett syndrome (CRS) from 2008 to 2022 (13). All participants with RTT were females, had a pathogenic *MECP2* variant and were aged six to 10 years at study entry. Assessments conducted before age 19 years were included in the dataset. Participants were excluded if they had only attended the CRS on one occasion and video data were excluded if gross motor video data were insufficient. For two children, videos were classified as insufficient because more than three items could not be coded. One visit shortly after a spinal fusion was excluded from analysis.

The Danish RTT database is authorized according to Danish legislations and approved by the Knowledge Center on Data Protection in the Capital Region of Denmark (VD-2019-131). The videos are saved for quality control with informed consent of the legal guardians.

### 2.2 Medical variables

Participants were evaluated by a pediatrician (GR, AM-B) at each visit and available data on comorbidities that are common in RTT were categorized as follows: Epilepsy (caregiver-report and review of medical files): never diagnosed, diagnosed - seizure free, diagnosed—seizure (monthly or less), diagnosed—seizure (weekly); autonomic respiratory dysfunction: none, minimal, intermittent, constant; and scoliosis: no, mild/moderate, severe, had spinal surgery. Medical files and videos were reviewed for individuals with on overall decline in RSGMS of four or more points for other indicators of health status or medical challenge: lower extremity bone fractures, surgeries other than spinal fusion, exacerbations of seizures which had led to a hospital contact, breathing dysrhythmias, pneumonias and movement disorders which could have adversely influenced on mobility. Medical records data and gross motor assessment data were collected on the same day.

### 2.3 Gross motor function

The RSGMS (14) was administered at each visit. The RSGMS is a validated RTT-specific outcome measure. Fifteen items describe sitting, standing, walking, and transfer skills and are rated on a four-point scale (no assistance (3), minimal assistance (2), moderate assistance (1), maximal assistance/unable (0)). A total score is calculated, and skills are further grouped into subscales of sitting (3 items), standing/walking (9 items), and challenge (3 items). The RSGMS has good support for its model fit and factor structure, and there is evidence of excellent test–retest reliability and known groups validity where scores were lower for older individuals with RTT and those with genetic variants usually associated with greater clinical severity (14). The minimal detectable difference (MDD) is four points on the total 45-point scale (14). Three researchers (MS, AKH, AMB) reviewed video recordings for completeness of gross motor skills data, scored each item and calculated total and subscale scores. After scoring, the previous video was viewed to confirm that changes in skill were observable and that coding rules were correctly applied. Three clinicians/physiotherapists/researchers scored the RSGMS independently after each visit and discussed scores if there were disagreements until consensus was reached to ensure consistency.

### 2.4 Statistical analysis

Descriptive statistics were reported as mean (SD), median (interquartile range [IQR]) or *n* (%) where appropriate to summarize the participant characteristics at their first visit. Total and subscale RSGMS scores were plotted for each participant to illustrate change over time, and a lowess (locally weight scatterplot smoothing) plot was included in each to show the overall trend. Additionally, selected individual RSGMS item scores and comorbidity categories at each assessment were tabulated. Two-level mixed-effects linear regression models with random intercepts and random slopes were used to analyze the effects of age on gross motor skills while accounting for within-subject correlations in repeated measures. In these models, unique identification numbers and age were specified as random-effects, with an independent covariance structure defining the variance–covariance structure of the random effects. Standard errors and 95% confidence intervals (CI) were controlled for participant clustering effects using a clustered sandwich estimator. Fixed effects variables of the models included age (centered around the global median value of 10 years and measured at five-year intervals), epilepsy, autonomic breathing difficulty, and scoliosis. Two multivariable modeling approaches were applied: Model 1 included age, variant, and one individual comorbidity variable at a time; Model 2 included age, variant, and all comorbidity variables. To examine the five-year age trends in scores by the severity of comorbidities, interaction terms between age and each comorbidity were examined. Specifically, in Model 1, the interaction was included directly between age and the single comorbidity variable being analyzed. In contrast, for Model 2, a series of iterative analyses were conducted, each adding an interaction term between age and one selected comorbidity variable at a time, while the remaining comorbidity variables were retained in the model as main effects without interaction. Adjusted means and the marginal changes over five-year age intervals by comorbidity severity were estimated using Stata’s margins command with the recycled predictions approach based on population-averaged (fixed-effects only) predictions from the mixed-effects models. Predictions were averaged across observed covariate values, and standard errors were derived using the delta method. Statistically significant results were defined as those with *p* < 0.05. All analysis was done using Stata version 16.1 (StataCorp. 2019. Stata Statistical Software: Release 16. College Station, TX: StataCorp LLC).

Medical data were reviewed for children with a decline of four or more points on the RSGMS since the previous observation, which is greater than the MDD value, to explore other potential explanatory variables.

## 3 Results

### 3.1 Baseline characteristics

**Figure 1:**
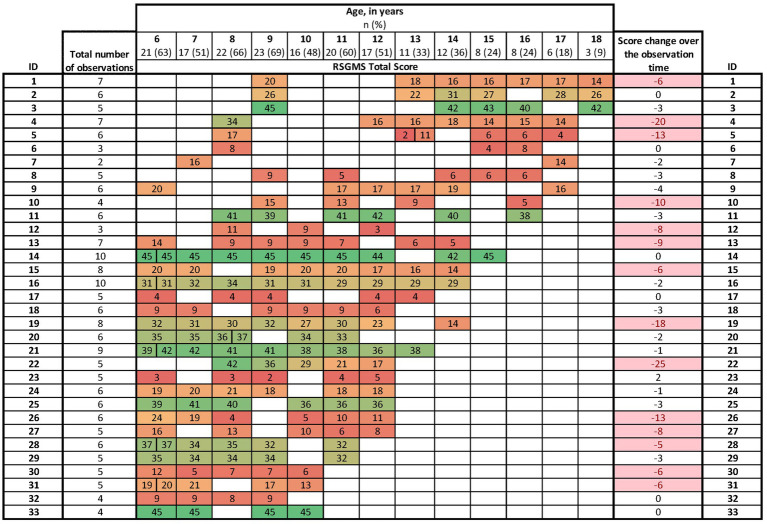
Age at first and follow-up assessments and corresponding RSGMS total score across the 191 observations in 33 children with Rett syndrome. RSGMS scores range from 0 to 45, with higher scores indicating better gross motor function. A color gradient from red (low scores) to green (high scores) is used to visualize the score distribution. Split cells represent repeated measurements at the same age. In the “Score change over the observation time” column, a decline of more than four points is highlighted in red.

The study population included 33 children with a mean age of 7.3 years (SD 1.2) (median 6.8 [IQR 6.4, 8.5]) at the first observation. The mean duration of follow-up was 6.8 years (SD 2.1), and the age at the last follow-up ranged from 9.3 to 18.7 years (mean 14.1 [SD 2.7]; median 13.8 [IQR 12.2, 16.6]). The children had two to 10 visits at CRS (mean 5.8 [SD 1.8]; median 6 [IQR 5, 6]). There was a total of 191 visits including 158 follow-up visits. Table 1 describes characteristics at first observation and all follow-ups. At the first observation, nearly a quarter of participants had the p. Thr158Met variant (*n* = 8, 24%), approximately two-fifths had never been diagnosed with seizures (*n* = 14, 42%) or with respiratory dysfunction (*n* = 12, 36%), and 32 (96%) had no or mild/moderate scoliosis. The mean RSGMS score was 24.0 (SD 13.2) out of a total of 45 at the baseline assessments. Figure 1 presents the pattern of follow-up observations, showing more observations available for adolescents than for middle childhood.

**Table 1:** Characteristics of the 33 children with Rett syndrome, at first observation and all follow-ups.

Variables	At first observation (*n* = 33)		At all follow-ups (*n* = 158)	
	Mean (SD) or *n* (%)	Median (IQR; range)	Mean (SD) or *n* (%)	Median (IQR; range)
Age, years	7.3 (1.2)	6.8 (6.4, 8.5; 6.1, 9.9)	11.5 (3.1)	11.2 (9.1, 13.8; 6.7, 18.7)
Duration of follow-up, years	6.8 (2.1)	6.9 (5.3, 8.1; 2.8, 11.2)	n/a	
Time between follow-ups, years	n/a		1.4 (1.2)	1.1 (1.0, 1.3; 0.4, 10.7)
Variant				
Large deletion	4 (12)		22 (13.9)	
p. Arg133Cys	1 (3)		4 (2.5)	
p. Thr158Met	8 (24)		46 (29.1)	
p. Arg168*	5 (15)		17 (10.8)	
p. Arg255*	1 (3)		2 (1.3)	
p. Arg270*	1 (3)		5 (3.2)	
p. Arg294*	1 (3)		4 (2.5)	
C-terminal deletion	4 (12)		23 (14.6)	
Other	8 (24)		35 (22.2)	
Epilepsy				
Never diagnosed	14 (42)		54 (34.2)	
Diagnosed—seizure free	7 (21)		28 (17.7)	
Diagnosed—seizure (monthly or less)	5 (15)		36 (22.8)	
Diagnosed—seizure (weekly)	7 (21)		40 (25.3)	
Respiratory dysfunction				
None	12 (36)		31 (19.6)	
Minimal	8 (24)		57 (36.1)	
Intermittent	13 (39)		66 (41.8)	
Constant	0 (0)		4 (2.5)	
Scoliosis				
No	16 (48)		43 (27.2)	
Mild/moderate	16 (48)		66 (41.8)	
Severe	1 (3)		22 (13.9)	
Had surgery	0 (0)		26 (17.1)	
Rett Syndrome gross motor scale				
Total score (0–45)	24.0 (13.2)	20 (14.35; 3.45)	22.3 (13.8)	19 (9.35; 3.45)
Sitting subscale (0–9)	8.5 (1.4)	9 (9.9; 3.9)	7.9 (1.8)	9 (7.9;2.9)
Standing/walking subscale (0–30)	13.8 (10.9)	11 (5.23; 0.30)	12.9 (11.1)	10 (0.23; 0.30)
Challenge subscale (0–6)	1.7 (1.9)	1 (0.2; 0.6)	1.5 (1.6)	1 (0.2; 0.6)

n/a, not applicable; IQR: interquartile range.

### 3.2 Trajectories of gross motor skills

**Figure 2:**
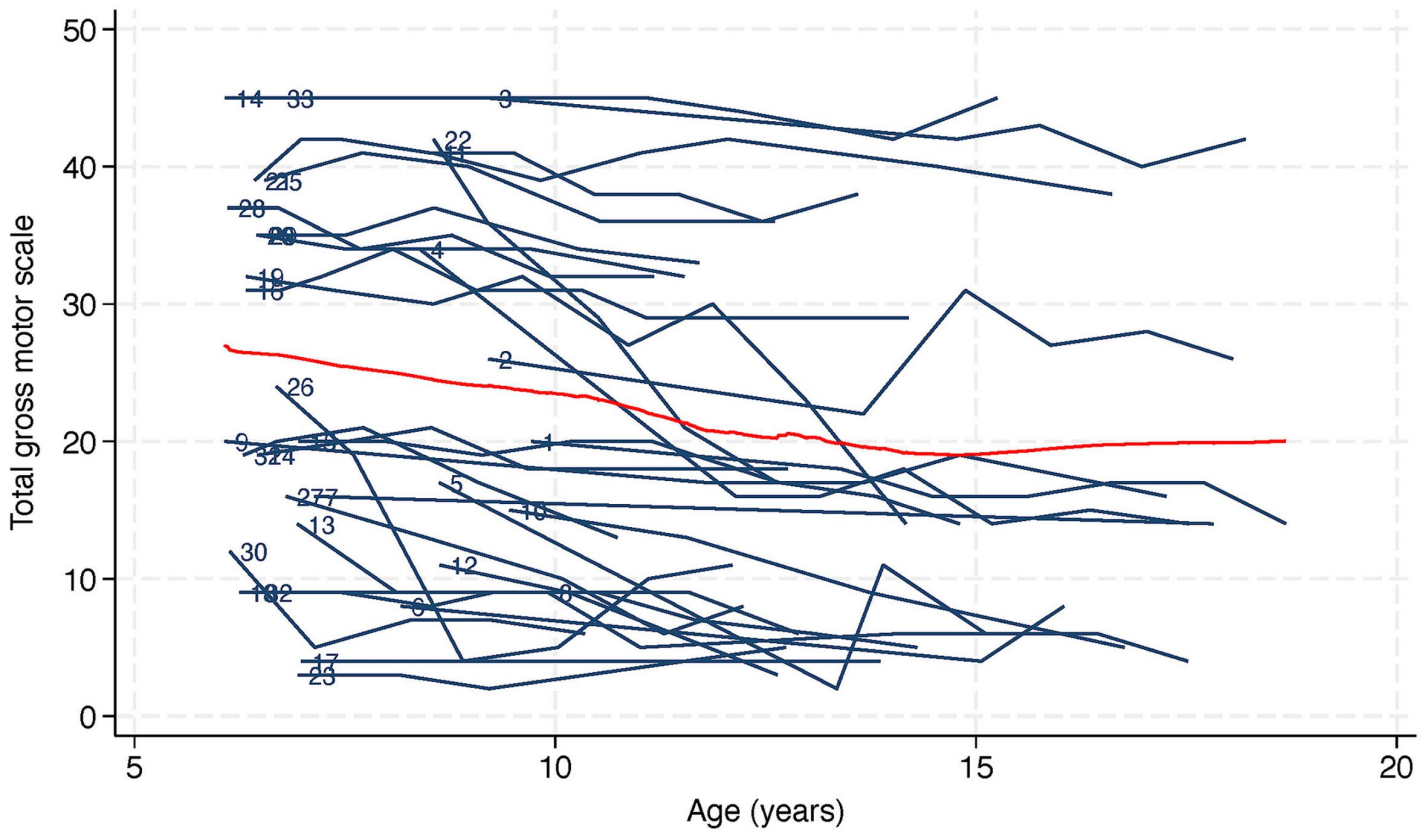
Trajectory and lowess plot of total gross motor scale in 33 children with Rett syndrome. Lowess, or locally weighted scatterplot smoothing, is represented as a red line on the plot. The numbers next to the trajectories indicate participant identification numbers.

**Figure 3:**
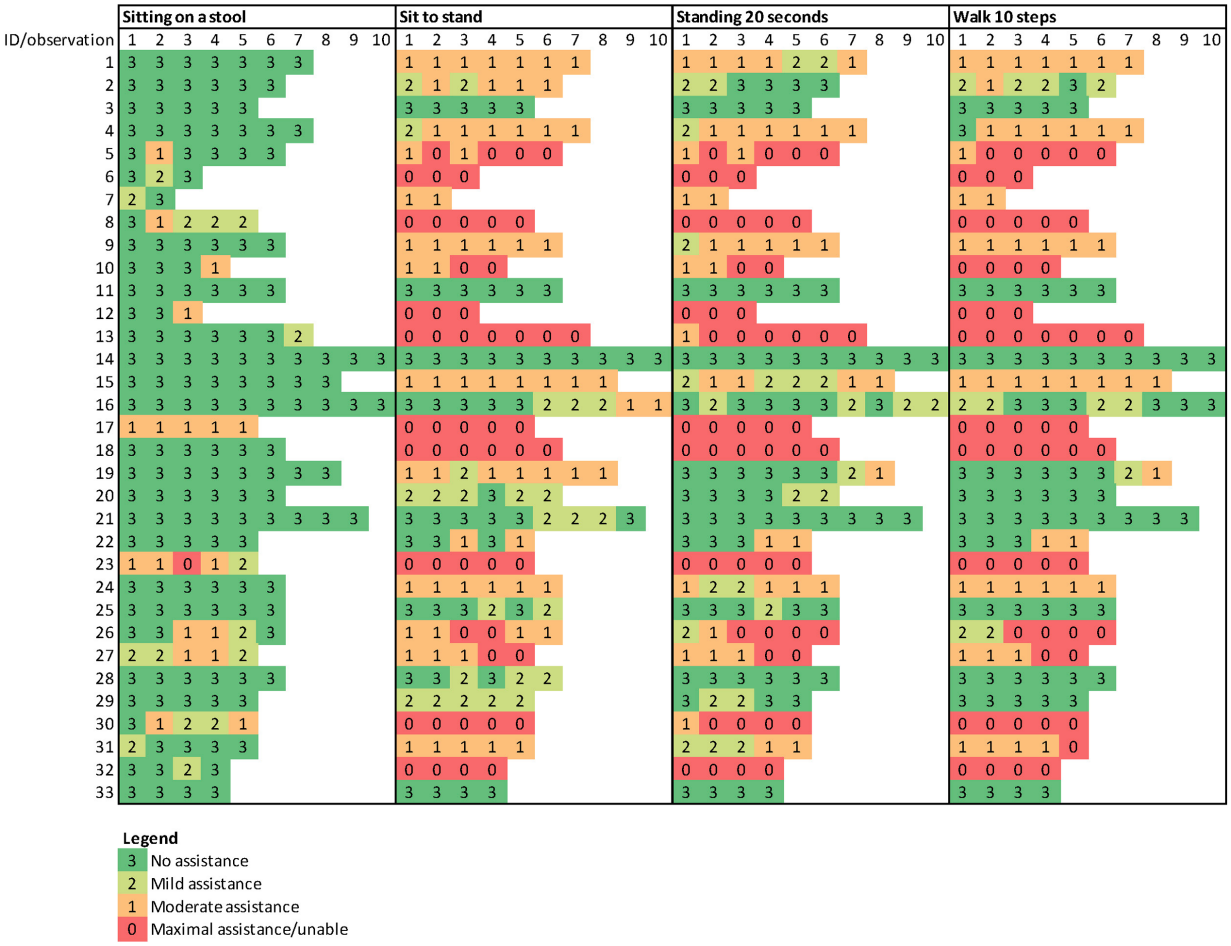
Trajectories of assistance level of selected gross motor items in 33 children with Rett syndrome.

**Figure 4:**
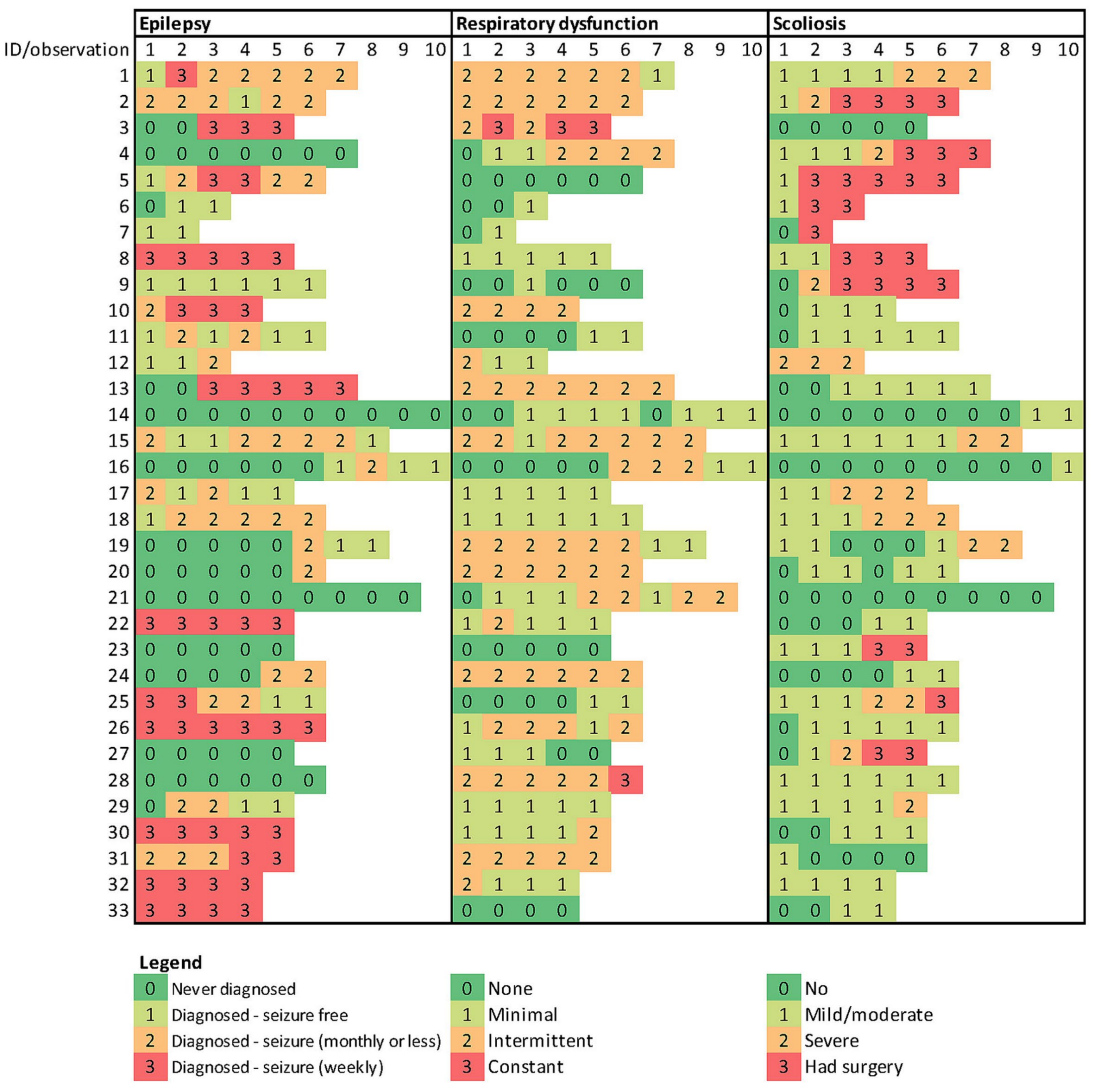
Trajectories of co-morbidities in 33 children with Rett syndrome.

Figure 2 shows a trajectory plot of the RSGMS score for each participant over time. Whilst scores for some individuals were stable, the lowess plot indicated a gentle decline in RSGMS scores from age six to 15 years. Figure 3 presents the scores for selected RSGMS items (related to sitting, transitions, standing and walking) where changing colours indicates changing scores. There are fewer rows where scores declined for the “sitting on a stool” and “walking 10 steps” items than for the “sit to stand” and “stand 20 s” items, suggesting that the sit to stand and standing task are more complex and difficult. Figure 4 indicates that the categories for epilepsy and autonomic respiratory dysfunction were severe at the last observation in approximately one-third of participants (weekly seizure: *n* = 10 [30%]; intermittent or constant autonomic respiratory dysfunction: *n* = 13 [39%]). On the other hand, scoliosis was severe at the last observation in 7 participants (21%) and 10 participants (30%) had undergone surgery.

From a total of 191 observations, after adjusting for age, variant, and all comorbidity variables (Model 2), total gross motor skills decreased by 4.0 points (95% CI −5.9, 2.1) per 5-year period (Table 2). The sitting, standing, walking, and challenge subscales decreased by 0.5 (95% CI −0.9, 0.0), 2.8 (95% CI −4.4, −1.2), and 0.6 (95% CI −1.0, −0.2), respectively (Table 3).

**Table 2:** Associations between total gross motor scale and age, variant and co-morbidities in 33 children with RTT.

	Model 1	Model 2
Number of observations (min, avg., max)	191 (2, 5.8, 10)	
	β (95% CI), *p*-value	
Age^1^ (every 5 years)	−4.3 (−6.0, −2.5), <0.01	−4.0 (−5.9, −2.1), <0.01
Variant		
Large deletion	Ref	Ref
p. Arg133Cys	−7.1 (−13.5, −0.8), 0.03	−8.2 (−14.6, −1.8), 0.01
p. Thr158Met	−3.8 (−12.2, 4.6), 0.38	−3.2 (−11.8, 5.5), 0.48
p. Arg168*	−15.0 (−21.6, −8.4), <0.01	−13.8 (−20.9,−6.8), <0.01
p. Arg255*	−14.8 (−21.2, −8.4), <0.01	−14.0 (−21.2, −6.8), <0.01
p. Arg270*	11.8 (5.5, 18.2), <0.01	12.5 (6.0, 19.0), <0.01
p. Arg294*	22.3 (15.9, 28.7), <0.01	21.5 (15.0, 28.0), <0.01
C-terminal deletion	12.1 (3.2, 21.0), 0.01	12.8 (4.1, 21.4), <0.01
Other	1.3 (−11.8, 14.5), 0.84	1.8 (−11.6, 15.1), 0.80
Epilepsy		
Never diagnosed or seizure free	Ref	Ref
At least monthly seizures	0.2 (−0.9, 1.3), 0.71	0.4 (−0.8, 1.6), 0.48
Autonomic respiratory dysfunction		
None or minimal	Ref	Ref
Intermittent or constant	0.4 (−1.8, 2.6), 0.70	0.2 (−1.7, 2.1), 0.87
Scoliosis		
No	Ref	Ref
Mild/moderate	−1.8 (−3.3, −0.2), 0.02	−1.9 (−3.6, −0.1), 0.03
Severe	−2.7 (−5.0, −0.3), 0.03	−2.6 (−4.9, −0.3), 0.03
Had surgery	−0.6 (−4.1, 2.9), 0.74	−0.6 (−4.1, 3.0), 0.80

β, beta coefficient; CI, confidence interval; Ref, reference level.

1 age centered at the global median of 10 years.

Model 1: each variable analyzed separately (age adjusted for variant; variant adjusted for age; each comorbidity adjusted for age and variant). Model 2: all variables included simultaneously (adjusted for age, variant, and all co-morbidities variables).

**Table 3:** Associations between gross motor subscales (sitting, standing/walking and challenge) and age, variant and co-morbidities in 33 children with RTT.

	Sitting		Standing/Walking		Challenge	
	Model 1	Model 2	Model 1	Model 2	Model 1	Model 2
Number of observations (min, avg., max)	191 (2, 5.8, 10)					
	β (95% CI), *P* value					
Age^1^ (every 5 years)	−0.8 (−1.1, −0.4), <0.01	−0.5 (−0.9, 0.0), 0.06	−3.0 (−4.4, −1.6), <0.01	−2.8 (−4.4, −1.2), <0.01	−0.5 (−0.9, −0.2), <0.01	−0.6 (−1.0, −0.), <0.01
Variant						
Large deletion	Ref	Ref	Ref	Ref	Ref	Ref
p. Arg133Cys	−0.4 (−0.7, −0.2), <0.01	−0.3 (−0.8, 0.2), 0.21	−6.5 (−12.3, −0.6), 0.03	−7.4 (−13.2, −1.6), 0.01	−0.6 (−1.2, 0.0), 0.07	−0.7 (−1.5, −0.0), 0.04
p. Thr158Met	−1.0 (−1.7, −0.3), <0.01	−0.9 (−1.6, −0.2), 0.01	−2.7 (−10.1, 4.7), 0.48	−2.2 (−9.9, 5.6), 0.58	−0.1 (−0.9, 0.6), 0.75	0.0 (−0.8, 0.7), 0.92
p. Arg168*	−2.6 (−3.9, −1.3), <0.01	−2.3 (−3.8, −0.8), <0.01	−11.4 (−17.4, −5.4), <0.01	−10.4 (−17.1, −3.8), <0.01	−1.0 (−1.6, −0.4), <0.01	−0.8 (−1.5, −0.2), <0.01
p. Arg255*	−1.8 (−2.1, −1.6), <0.01	−1.7 (−2.4, −1.0), <0.01	−12.0 (−17.9, −6.2), <0.01	−11.3 (−18.2, −4.5), <0.01	−1.0 (−1.6, −0.4), <0.01	−0.8 (−1.4, −0.1), <0.01
p. Arg270*	−0.2 (−0.4, 0.1), 0.15	−0.1 (−0.7, 0.4), 0.63	10.4 (4.5, 16.2), <0.01	10.9 (4.8, 16.9), <0.01	1.6 (1.0, 2.2), <0.01	1.7 (1.0, 2.3), <0.01
p. Arg294*	0.4 (0.1, 0.7), <0.01	0.1 (−0.5, 0.7), 0.76	17.8 (11.9, 23.6), <0.01	17.2 (11.3, 23.1), <0.01	4.5 (3.9, 5.1), <0.01	4.5 (3.8, 5.1), <0.01
C-terminal deletion	−0.2 (−0.5, 0.2), 0.40	−0.2 (−0.8, 0.4), 0.47	10.9 (2.7, 19.1), <0.01	11.5 (3.4, 19.7), <0.01	1.4 (0.7, 2.1), <0.01	1.6 (0.9, 2.3), <0.01
Other	−1.3 (−2.6, 0.0), 0.05	−1.2 (−2.6, 0.2), 0.10	1.5 (−9.2, 12.2), 0.78	1.9 (−9.0, 12.9), 0.73	1.2 (−0.5, 3.0), 0.17	1.4 (−0.4, 3.1), 0.13
Epilepsy						
Never diagnosed or seizure free	Ref	Ref	Ref	Ref	Ref	Ref
At least monthly seizures	−0.2 (−0.7, 0.3), 0.37	−0.2 (−0.8, 0.3), 0.39	0.3 (−0.8, 1.3), 0.62	0.4 (−0.6, 1.4), 0.41	0.1 (−0.1, 0.2), 0.45	0.1 (−0.1, 0.3), 0.38
Autonomic respiratory dysfunction						
None or minimal	Ref	Ref	Ref	Ref	Ref	Ref
Intermittent or constant	−0.1 (−0.7, 0.6), 0.84	−0.1 (−0.7, 0.6), 0.86	0.5 (−1.2, 2.2), 0.57	0.2 (−1.2, 1.7), 0.73	0.2 (−0.0, 0.5), 0.06	0.2 (0.0, 0.4), 0.07
Scoliosis						
No	Ref	Ref	Ref	Ref	Ref	Ref
Mild/moderate	−0.3 (−0.7, 0.1), 0.16	−0.3 (−0.8, 0.2), 0.30	−1.4 (−2.5, −0.2), 0.02	−1.4 (−2.7, −0.1), 0.03	−0.2 (−0.4, 0.0), 0.07	−0.2 (−0.4, 0.0), 0.07
Severe	−0.6 (−1.3, 0.1), 0.10	−0.6 (−1.4, 0.1), 0.11	−2.1 (−4.1, −0.2), 0.03	−2.1 (−4.0, −0.2), 0.03	−0.1 (−0.4, 0.2), 0.55	−0.1 (−0.4, 0.2), 0.69
Had surgery	−0.8 (−1.8, 0.2), 0.12	−0.8 (−1.8, 0.2), 0.11	−0.2 (−3.4, 3.1), 0.92	−0.1 (−3.4, 3.1), 0.94	0.2 (−0.3, 0.7), 0.38	0.2 (−0.2, 0.7), 0.34

β, beta coefficient; CI, confidence interval; Ref, reference level.

1 age centered at the global median of 10 years.

Model 1: each variable analyzed separately (age adjusted for variant; variant adjusted for age; each comorbidity adjusted for age and variant). Model 2: all variables included simultaneously (adjusted for age, variant, and all co-morbidities variables).

### 3.3 Factors influencing trajectories of gross motor skills

Compared to the large deletion variant, mean total RSGMS scores were lower for the p. Arg133Cys (*β* coefficient −8.2, 95% CI − 14.6, −1.8), p. Arg168* (β coefficient −13.8, 95% CI − 20.9, −6.8) and p. Arg255* (β coefficient −14.0, 95% CI − 21.2, −6.8) variants, but higher for the p. Arg270* (*β* coefficient 12.5, 95% CI 6.0, 19.0), p. Arg294* (*β* coefficient 21.5, 95% CI 15.0, 28.0) and C-terminal deletion (β coefficient 12.8, 95% CI 4.1, 21.4) variants. Compared to no seizures or no or minimal respiratory dysfunction, having any seizures or intermittent or constant autonomic respiratory dysfunction had little effect on the mean total RSGMS score. Compared to no scoliosis, mean total scores were lower in those with a mild/moderate (*β* coefficient −1.9; 95% CI −3.6, −0.1) or severe (β coefficient −2.6; 95% CI −4.9, −0.3) scoliosis but mean scores were similar for those who had scoliosis surgery (β coefficient −0.6; 95% CI −4.1, 3.0) (Table 2). Similar patterns were observed for the subscale scores (Table 3).

Table 4 presents the five-year age trends by comorbidity severe level for total and subscale scores. Adjusting for age, genetic variant and all comorbidities, all severity levels of epilepsy, autonomic breathing dysfunction and scoliosis were associated with a decreasing five-year age trend of the total scale score and the standing/walking subscale score, except for the group with surgically corrected scoliosis. In contrast, the age trends for surgically corrected scoliosis were positive although not statistically significant, suggesting very small improvements in gross motor skills. For the sitting subscale, the age trend decreased the most when severe scoliosis was present (estimated marginal change −1.9; 95% CI −3.3, −0.5) and the least when no scoliosis was observed (estimated marginal change −0.1; 95% CI −0.3, 0.2). The challenge subscale generally decreased over time regardless of severity, with surgically treated scoliosis having the smallest decline over time (estimated marginal change −0.3; 95% CI −0.9, 0.3).

**Table 4:** Five-year age^1^ trends of the total gross motor scale and gross motor subscales (sitting, standing/walking and challenge) by co-morbidities severity in 33 children with RTT.

	Total		Sitting		Standing/walking		Challenge	
	Model 1	Model 2	Model 1	Model 2	Model 1	Model 2	Model 1	Model 2
Number of observations (min, avg., max)	191 (2, 5.8, 10)							
	Estimated marginal change (95% CI), *P*-value							
Epilepsy								
Never diagnosed or seizure free	−4.3 (−6.0, −2.6), <0.01	−4.0 (−5.9, −2.1), <0.01	−0.5 (−0.9,−0.2), <0.01	-0.2 (−0.6, 0.2), 0.31	−3.2 (−4.6, −1.8), <0.01	−3.1 (−4.7, −1.5), <0.01	−0.5 (−0.8, −0.2), <0.01	−0.6 (−0.9, −0.2), <0.01
At least monthly seizures	−4.3 (−6.6, −2.1), <0.01	−3.9 (−6.2, −1.7), <0.01	−1.0 (−1.6, −0.5), <0.01	−0.8 (−1.5, −0.1), 0.03	−2.8 (−4.6, −0.9), <0.01	−2.5 (−4.4, −0.6), <0.01	−0.5 (−1.0, −0.1), 0.02	−0.6 (−1.1, −0.1), 0.02
Autonomic respiratory dysfunction								
None or minimal	−5.6 (−8.4, −2.8), <0.01	−5.2 (−7.9, −2.4), <0.01	−0.7 (−1.2, −0.3), <0.01	−0.4 (−0.9, 0.1), 0.10	−4.1 (−6.5, −1.8), <0.01	−3.9 (−6.2, −1.5), <0.01	−0.6 (−1.0, −0.2), <0.01	−0.6 (−1.0, −0.2), <0.01
Intermittent or constant	−2.5 (−4.4, −0.5), 0.01	−2.5 (−4.4, −0.7), <0.01	−0.8 (−1.4, −0.2), <0.01	−0.5 (−1.2, 0.1), 0.11	−1.4 (−2.9, 0.2), 0.08	−1.5 (−3.0, −0.0), 0.05	−0.5 (−0.9, −0.1), 0.01	−0.6 (−1.0, −0.2), <0.01
Scoliosis								
No	−4.9 (−7.2, −2.5), <0.01	−5.0 (−7.3, −2.7), <0.01	−0.3 (−0.5, −0.0), 0.05	−0.1 (−0.3, 0.2), 0.49	−3.6 (−5.5, −1.7), <0.01	−3.8 (−5.6, −1.9), <0.01	−0.6 (−1.3, 0.1), 0.07	−0.7 (−1.4, 0.0), 0.06
Mild/moderate	−4.4 (−6.7, −2.1), <0.01	−4.5 (−6.8, −2.2), <0.01	−0.5 (−1.2, 0.2), 0.19	−0.4 (−1.1, 0.2), 0.20	−3.3 (−5.4, −1.2), <0.01	−3.4 (−5.5, −1.2), <0.01	−0.6 (−1.0, −0.2), <0.01	−0.6 (−1.0, −0.3), <0.01
Severe	−5.3 (−8.4, −2.3), <0.01	−5.2 (−8.2, −2.2), <0.01	−1.8 (−3.4, −0.3), 0.02	−1.9 (−3.3, −0.5), 0.01	−3.6 (−6.4, −0.7), 0.01	−3.4 (−6.1, −0.6), 0.02	−0.5 (−0.9, −0.2), <0.01	−0.4 (−0.8, −0.1), <0.01
Had surgery	0.7 (−2.0, 3.4), 0.61	0.7 (−2.0, 3.5), 0.60	−0.6 (−1.5, 0.2), 0.16	−0.6 (−1.5, 0.3), 0.19	1.6 (−0.9, 4.1), 0.21	1.7 (−0.9, 4.2), 0.20	−0.3 (−0.9, 0.3), 0.33	−0.3 (−0.9, 0.3), 0.34

β, beta coefficient; CI, confidence interval.

1 age centered at the global median of 10 years.

Model 1: adjusted for age and variant.

Model 2: adjusted for age, variant and all co-morbidities variables.

### 3.4 Potential factors that explained decline in gross motor skills

Fourteen (42%) girls had a decline of four or more points on the RSGMS over the observation time, greater than the MDD value (Figure 1). Reviews of medical records and videos suggested possible explanations such as increasing muscle stiffness, hypotonia and movements disorders (such as stereotypies, tremor, myoclonus, ataxia, and dystonia, *n* = 12), hip, hamstrings or Achilles tendon surgeries or fractures (*n* = 7) and worsening of comorbidities such as seizures (*n* = 10). Interestingly, only three of these 14 girls had had spinal surgery. Six (ID 5, 13, 19, 26, 30 and 31) of the 14 girls had more rapid changes, observed from one visit to the next (Figure 1), with four or more points decline over 12–19 months (mean 14 months). Sudden declines were associated with increased seizure activity (*n* = 3), severe apnoea or hypoventilation (*n* = 3), orthopaedic surgeries or fractures (*n* = 3), worsening of movement disorder such as dystonia (*n* = 2) and recurrent pneumonias (*n* = 1). Table 5 presents case studies describing the clinical history of three girls with RTT with a stable, gently declining, or more rapidly declining gross motor trajectory.

**Table 5:** Case studies of girls with Rett syndrome.

Case 1: A girl with a stable gross motor course and two scoliosis surgeries (ID 2)	A stable gross motor phenotype was documented at six assessments from age 9 to 18 years.
	She was born at a gestational age of 34 weeks, and the neonatal period was uncomplicated. Her development was slightly delayed; she learnt to crawl by 12 months, walk by 21 months, use a pincer grasp by 19 months, and could say approximately 15 words by 16 months of age. Around the age of 2 years, she lost words and hand skills, ceased crawling, and lost the ability to transfer from sitting to standing although she remained able to walk. Her eye contact deteriorated, and she developed screaming attacks. The suspicion of Rett syndrome was raised at age 2.5 years due to this regression and the development of hand stereotypies. The clinical diagnosis was confirmed genetically, revealing a large deletion of exons 3 and 4 in *MECP2*.
	Her eye contact improved after the regression period, but she did not regain use of words and hand skills. At age 4 years, she was treated with antiepileptic medication for a short period due to absences and the suspicion of epilepsy. There has been no concern regarding epilepsy since then. Over the years, she has experienced breath-holding and hyperventilation. During childhood, she easily became frustrated, had screaming attacks and experienced poor sleep.
	She developed scoliosis, for which she underwent spinal fusions at age 14.5 years (TH4-L1) and 16.5 years (TH12-L5). After the first surgery, she experienced less pain, reduced vomiting, and a more stable breathing pattern. Following the second surgery, she slept better, her mood improved, and she became more patient.
	Her RSGMS total score remained the same at the 9- and 18-year assessments (26/45 points) although it fluctuated in the intervening years, with decreasing scores before the spinal fusions and increasing score after the first surgery.
	At the age of 26, she remains ambulant and experiences a calmer mood compared to during her childhood.
Case 2: A girl with a gradual gross motor decline and movement disturbances (ID 1)	This case study presents a girl who was evaluated seven times from the age of 9 to 18 years and exhibited gently declining gross motor skills.
	Her birth and neonatal period were uncomplicated. During the first 6 months, she was easy to handle, difficult to feed and had low muscle tone. At 9 months, she was admitted to hospital due to persisting vomiting over a period of 1.5 months, but no indication for further investigations or treatment was found.
	By 18 months, her development raised concerns; she did not crawl and was uninterested in social interactions. At 30 months, she lost hand skills and was unable to finger feed, concurrently developing hand stereotypies. She also lost the few words she had learnt, and her eye contact was poor. At 3 years old, she learnt to walk with support. When nearly 3 years of age, she was admitted to hospital for investigations due to the developmental concerns, suspected seizures and observations of irregular breathing. Genetic testing confirmed the suspicion of RTT, revealing the variant p. T158M in *MECP2*.
	Her medical history included frequent irregular breathing and epilepsy treated since the age of three. Unequal leg length and scoliosis were observed from the age of 7 years. Her Cobb angle reached 70 degrees and her scoliosis was managed conservatively.
	RSGMS scores declined steadily from 20/45 to 14/45 over 9 years. It became increasingly difficult for her to sit on the floor, stand and walk, requiring two-handed support by the end of the study period. Video review indicated worsening of balance in standing and walking and transferring from standing to sitting became more difficult due to body stiffness.
	Dystonic movements increased during adolescence and have persisted to her current age of 26 years, accompanied by breath holding attacks. Despite these challenges, she remains able to walk with assistance.
Case 3: A girl with a sudden gross motor decline, movement disorders and epilepsy (ID 26)	This case study presents a girl who was evaluated six times from the age of 6 to 12 years and experienced sudden gross motor declines.
	Her birth and neonatal period were uncomplicated, and she was a happy and content baby who ate and slept without problems. Her development was satisfactory the first 12 months; she could sit at 7 months, and crawl and stand with support at 12 months. However, at 14–15 months her parents became concerned as her development had ceased progressing, and she had lost hand skills. Her eye contact deteriorated, and she no longer smiled as she used to. The signs of Rett syndrome likely began shortly after her first birthday including poorer balance. At 16 months, she was evaluated by a pediatrician who suspected Rett syndrome and the variant p. Thr158Met in *MECP2* was identified. She was referred to physiotherapy; her balance improved, and she learned to walk with support. Her eye contact also improved. At her first visit at CRS at 19 months, she could walk with support and crawl although her hand-mouth stereotypies interfering with her ability to achieve the tasks.
	She developed epilepsy before age 2 years, experienced autonomic dysfunction affecting breathing and gastrointestinal function, and developed a mild scoliosis. At 7 years old, she underwent gastrostomy insertion. Her seizures significantly impacted her everyday life and were difficult to treat. By age 8 years, and around her third visit at CRS, her daily seizures were additionally treated using a nervus vagus stimulator. During the visit, she also exhibited considerable body restlessness with stereotypies and a dystonic- and chorea-like movement pattern. By 11 years, at her fifth visit, her epilepsy had improved, and she was less restless.
	Her RSGMS score decreased by 20 points from age 6.7 to 8.9 years primarily due to declines in the “standing and walking” subscale. Her balance worsened during the early part of the study period. Additionally, her stereotypies, fluctuating muscle tone and movement disturbances impaired her standing and walking abilities. Fluctuations appeared to coincide with the stability of medical symptoms.
	Currently, at age 14 years, she is primarily wheelchair-bound but she can walk short distances with support. Her medical conditions are stable.

## 4 Discussion

Knowledge of natural history is an important component of clinical trial readiness and informs clinical care to support gross motor function (10, 19). Using a validated measure of gross motor function in RTT, we documented a gentle decline in overall gross motor skills in 33 children with RTT. For nearly half of the participants, declines appeared to be accelerated by acute medical episodes such as refractory seizures or fractures. This new knowledge of the natural history of gross motor skills in RTT is important for interpretating findings from clinical trials and could be compared with outcomes in clinical trials testing new therapeutics.

RTT is uniquely characterized by a period of developmental regression during early childhood, classically described as loss of hand and/or communication skills, altered gait, and development of hand stereotypies (5). Beyond the regression period, longitudinal data from the US Natural History has demonstrated increasing clinical severity with increasing age and that ability to walk closely paralleled the trajectory of overall clinical severity (20), consistent with the earlier descriptions of poorer gross motor function in adulthood (21). Building on this foundational work, we have used a validated gross motor assessment to map gross motor skill trajectories at a more granular level (14). We were surprised to find that the average gross motor score declined 4 points every 5 years, a slightly higher rate of decline than the average decline of 3.4 points every 5 years that we previously reported for adults with RTT (13). These previous findings might have been caused by a healthy survivor effect in the adult group.

For specific skills, declines in sitting and walking were less frequent than declines for sit-to-stand and standing skills, suggesting that transitions and balance are complex skills that are more vulnerable to loss in Rett syndrome. A qualitative mapping study of the regression period illustrated poorer balance when sitting, standing and walking in many children (22). We now report that balance is an ongoing gross motor difficulty. It was exemplified in case 3, a girl who was challenged by poor balance in the regression period as well as later in childhood. In our longitudinal evaluation of hand function skills, approximately one third lost some hand function across 3 year observation periods, especially younger children after the regression period and often associated with decline in ambulation (23). Both gross motor and hand function skills are fundamentally changed following regression in RTT and may continue to decline during childhood.

There was variation in RSGMS scores by the type of genetic variant. Higher scores were observed in individuals with the p. Arg294* variant, consistent with previous cross-sectional RSGMS data (14). However, the finding for p. Arg133Cys in this study (lower scores) conflicts with previous literature showing higher scores, likely reflecting the single participant with this variant in our sample. We adjusted for genetic variant in our longitudinal models although our sample size precluded capacity to evaluate trajectories by genetic variant. Epilepsy, autonomic respiratory dysfunction, and scoliosis, except for surgically managed scoliosis, were each associated with similar changes in RSGMS scores. There is usually recovery of gross motor skills back to pre-operative levels following spinal fusion (24). Our data suggested that the spinal stability afforded by spinal fusion could have a protective effect on gross motor skills where skills were regained and maintained. The girl in case 1 showed a decrease in RSGMS scores before her scoliosis surgeries and an increase after the first surgery. Concurrently, her health improved with less pain and vomiting, a more stable breathing pattern and better sleep.

We analyzed medical records to identify medical events that could explain declines in scores greater than the MDD value of 4 points since the previous observation. Some declines were potentially related to acute changes in muscle tone, movement disorder or seizures. The girls in case 2 and 3 illustrate the potential roles of difficult to manage seizures, changes in muscle tone and movement disorder that appeared to adversely affect gross motor skills and wellbeing. Using the Rett Syndrome Rigidity Distribution score, Humphreys et al. observed rigidity in most individuals which could appear as early as 3 years, progressed over time and was more apparent in non-ambulatory individuals (18). Movement disorder symptoms are well established in RTT and measurement using a validated scale could complement other outcome measures in clinical trials. For others in the study, musculoskeletal trauma associated with surgery or fractures could have hastened declines, illustrating the fragility of gross motor status in the wake of enforced periods of inactivity. Establishing goals for increased physical activity and reduced sedentary behaviors should be considered as soon as the individual is medically stable (25, 26). We were not able to include information regarding dysphagia or difficulties in feeding which might have influenced respiratory health and overall wellness (27).

The cohort was molecularly and clinically well characterized. A validated gross motor scale was used, skills were videotaped to enable ongoing review of data, three experienced clinicians/research coded the videos independently and then conferred to ensure consistency of coding. Observations from clinic visits over periods of up to 11 years were captured. We acknowledge that our sample size was small, and conclusions regarding genetic variant effects should be interpreted cautiously due to limited numbers within individual variant groups, particularly p. Arg133Cys, p. Arg255*, p. Arg270*, and p. Arg294* (each *n* = 1). We could not examine trajectories by genetic variant, and two of the most common eight variants were not represented (p. Arg106Trp and p. Arg306Cys). Medical records data were available to describe common comorbidities. However, evaluations of scoliosis were based on clinical examinations not involving radiological assessments and changing clinical features such as changes in muscle tone or movement disorder or pneumonia were described but concordance with standardized definition was not established. Rather, the medical records were considered an important practice resource that could suggest factors that could adversely influence gross motor function, and which should be studied further. Another limitation is that changes in medication that might have influenced balance and motor abilities were not captured. We acknowledge that other factors could influence the trajectory of gross motor skills such as sleep quality and the role of physical activity or skills practice, nor did we score movement disorders systematically. However using a validated measure of gross motor function, we identified novel information about the natural history of gross motor skills in children, enabling comparison with previously examined adult trajectories and potentially providing natural history data for comparison with treatment groups receiving new therapeutics such as gene therapy. Ongoing studies are needed to replicate the findings with larger sample sizes and to further examine trajectories for individual genetic variants, medical complexities and different amounts of skills practice and physical activity. Evaluations of how to maintain and improve motor skills over time in RTT are important topics for research.

## Data Availability

The datasets presented in this article are not readily available because of ethical and privacy restrictions. Requests to access the datasets should be directed to the corresponding author.
